# Do we need special pedagogy in medical schools? – Attitudes of teachers and students in Hungary: a cross-sectional study

**DOI:** 10.1186/s12909-020-02385-x

**Published:** 2020-11-26

**Authors:** Zsuzsanna Varga, Zsuzsanna Pótó, László Czopf, Zsuzsanna Füzesi

**Affiliations:** 1 Department of Behavioral Sciences, University of Pécs Medical School, Szigeti str. 12, Pécs, 7624 Hungary; 2 1st Department of Medicine, University of Pécs Medical School, Ifjúság str. 13, Pécs, 7624 Hungary

**Keywords:** Learning outcomes, Medical teacher, Medical education, Pedagogical skills, Gap matrix

## Abstract

**Background:**

The quality of medical education is a key factor. The fact that mostly physicians teach tomorrow’s physicians without acquiring pedagogical skills before becoming a teacher is a cause of concern. In Hungary, where traditional teaching methods are common, and teachers have not had pedagogical courses in medical education there has not been any research dealing with the issue. On the one hand, we aimed with this cross-sectional study to examine the attitudes of teachers towards learning outcomes of medical students to get a view about the opinions about their importance and rate of delivery. On the other hand, we analyzed the pedagogical skills of teachers from the students’ and teachers’ perspective in Hungary.

**Methods:**

Data collection through self-reported questionnaires in online form in all the four Hungarian higher education institutions offering medical education was carried out among teachers and students with active student legal status in 2017. We validated the questionnaires of the two respondents’ groups. We used gap matrices to represent the correspondences of the delivery and perceived importance of the learning outcomes. We calculated averages of the pedagogical skills and compared them with t-tests.

**Results:**

The response rates are 11.18% in case of the students (1505) and 24.53% in case of the teachers (439). The results indicate the lack of concordance between the rates of the learning outcomes in terms of their importance and delivery - no positive gap can be observed -, and the need for pedagogical skills among teachers and students. The opinions of students compared to teachers’ are all statistically higher according to the averages.

**Conclusions:**

The study results underline the necessity of a transition and paradigm shift in medical education from delivering solely professional knowledge towards pedagogically prepared practice and patient oriented teaching methods as well as acquiring pedagogical knowledge as part of the training of medical teachers in Hungary.

## Background

The quality of medical education is a key factor in securing future professional medical care, with the background of the global changes in medical knowledge and work regarding the fact that today’s physicians teach tomorrow’s physicians. Being expert in teaching, acquiring pedagogical knowledge and using teaching methods consciously can be beneficial for both parties - teachers and students - taking part in medical education. Based on some previous research comparing different types of pedagogical methods we believe that we can serve our students best by fusing elements of various traditional - such as lectures, if they refer to the traditional picture of a professor standing in front of and talking at a large group of students who are passively absorbing information - and non-traditional - such as team-based learning, case-based learning and interactive large-group learning sessions which include student engagement - pedagogical methods [1, 2].

International research suggests that using certain types of non-traditional methods may particularly be beneficial for students with lower academic performance. For example, in the study of Krupat at all [2] where students taking part in the research (and learning with case-based collaborative learning method) scored higher on examinations than those where traditional methods were used. Then the question arises: Why do teachers use traditional teaching methods in a wider range in medical education in Hungary, when several feedbacks given by students and findings results suggest the introduction and use of non-traditional teaching methods [1–6]?

To get answers to this question we aimed to analyze the attitudes towards the learning outcomes (which are determined by law for completion and exit requirements in Hungary and summarize the key knowledge, skills and attitudes medical students are expected to acquire by the time they qualify as doctors) and the pedagogical skills (which include professional, didactic, communicational, psychological knowledge, organizing and leading the learning process, improving adapting skills, decision making skills, empathy, learning the ability of professional cooperation [5, 6]) since teachers’ attitudes, teaching experience and practices often shape their behavior during the courses acting as a teacher and a clinician-educator [7]. The attitude of “I do not need to know anything about educational theory to be a good teacher.” [7] is no longer the way how teachers should approach teaching [7]. They must be able to use different types of educational tools along with the different learning methods [8]. Unfortunately, teachers participating in medical education are rarely experts in pedagogy. They usually acquire teaching skills based on their experience as learners and on the basis of their personal experience and observations [8, 9] that is the reason scientific literature is pointing to the inadequacy of the pedagogical knowledge of medical teachers [10–12]. In line with this, several research studies have been conducted in this field and directed attention to the need for reform of medical education [13] and a greater awareness of the requirement to train medical doctors as educators was raised, and special pedagogy courses have been set up to satisfy the needs of the students [14] and to ensure that besides their professional medical knowledge and skills they must guarantee teaching excellence [15–17].

In fact, Dent and Harden reviewed the topic of medical staff development extensively in the Practical Guide for Medical Teachers [18] and have been already put into practice in some medical schools (such as Duke–NUS Medical School, Nanyang Technological University, Harvard University), but not in Hungarian medical schools (with the exception of some project works such as an e-learning module prepared and financed by TÁMOP-4.1.1.C-13/1.).

The objective of our study was to get a deeper view with the method of gap analysis matrices on the attitudes of teachers and students in Hungary where non-traditional teaching methods are rarely used (except – clinical and other - practices), most teachers teach in lecture format with presentations. In our survey we asked the teachers whether they felt equipped to deliver the learning outcomes prescribed for medical student in Hungary to have a view on the effectiveness of traditional teaching methods. Besides we focused on the need for further development of teachers’ pedagogical skills. Our intent was to raise the awareness for the change from teacher- to student-oriented teaching methods [19–23] that is the reason we analyzed the teachers’ attitudes towards the learning outcomes of the general medicine major and the teachers’ and students’ pedagogical skills used by teachers in Hungary, using a national survey in an international setting. Our hypotheses were – based on our previous analyses dealing with students’ satisfaction and students’ feedbacks on teaching [24–26] – that teachers realize the learning outcomes of the profession of doctors more important than they focus on teaching them during their courses. The second one was that teachers estimate the importance of further development of the pedagogical skills less than students in Hungary.

## Methods

### Study design, questionnaire

Since there have not been any systematic studies analyzing the attitudes towards the learning outcomes and pedagogical skills in Hungarian medical schools therefore, we used self-developed online surveys in Hungarian, English and German language resulted a cross-sectional database. The study period was between 2017 November 9 to December 28. The four higher education institutions offering medical education in Hungarian, in English and in German are as follows: Semmelweis University, Faculty of Medicine, University of Debrecen Faculty of Medicine (except German programme), University of Pécs, Medical School, University of Szeged Faculty of Medicine. In all four institutions international students are welcomed, the 12-semester training period is divided into two parts. The first part consists of a two-year preclinical study period in the basic sciences; the second part is focused on clinical studies and lasts for four years. The internship period takes place during the rotational year and is generally spent at University clinics or hospitals.

We developed two questionnaires, one for teachers and one for students in more phases. We constituted a committee of experts (dealing with educational matters, quality assurance, sociologists, linguists, representatives of the students) who took part in the development of the surveys.

In the first phase, we analyzed the learning outcomes listed in the Hungarian law for completion and exit requirements. In the second phase, we performed semi-structured interviews among fifteen teachers to get information about their teaching methods, the need for educational courses and their attitudes towards the learning outcomes listed in the Hungarian law for completion and exit requirements. Based on the first two phases – in the third phase – we generated the items and set up two dimensions of the questions to be able to use the gap matrix during the analysis. We selected thirty-one learning outcomes and defined the questions in terms of their importance and the rate of delivery. In case of the pedagogical skills we focused on the questions whether teachers need further educational training from the perspectives of the students and teachers based on pedagogical skills for the teacher’s profession (listed in Table 4, Hegyi’s, Ballér’s grouping).

In sum, we asked questions regarding the importance, the rate of delivery, the rate of acquisition of the learning outcomes and the need for further development of teachers’ pedagogical skills (Additional file 1, Additional file 2). From teachers’ and students’ perspective the following questions are considered relevant to this article:

1. How important do you consider the listed learning outcomes to perform your everyday job as a doctor? (teachers)
2. To what extent do you deliver the listed learning outcomes during your courses? (teachers)
3. To what extent do you think you need further development in the following pedagogical skills? (teachers)
4. What do you think in which pedagogical skills would be needed further education for the teachers in your Faculty? (students)

We used quantitative content validity method in the fourth phase. According to the evaluation of our panel of experts the CVR [27] was calculated for the chosen thirty-one items (in connection with the learning outcomes), for twenty-four the CVR was bigger than 0.49, so we kept those in the questionnaires (Table 1).

**Table 1:** The learning outcomes of physicians with their importance and delivery rates used in our questionnaires by the teachers

	Importance			Delivery		
	N	Mean	Std. Deviation	N	Mean	Std. Deviation
1. The timely theoretical and practical knowledge to the everyday work	438	4.77	0.49	420	4.26	0.85
2. The professional practice needed for the everyday work	437	4.91	0.35	364	3.55	1.12
3. The knowledge of historical overview of the medical disciplines	438	2.78	1.05	359	2.73	1.20
4. The flexible professional and everyday thinking	437	4.78	0.48	400	3.87	0.91
5. Respecting human dignity of the patients and the relatives during patient care	436	4.85	0.44	298	3.64	1.33
6. Respecting the different demographic (sex, age), social and economic characteristics during patient care	437	3.98	1.11	301	3.38	1.35
7. Respecting individual specialty during patient care (e.g. familiar background, emotional state, sexual orientation)	437	4.19	0.96	294	3.37	1.32
8. Treating the emotional reactions of the patients and the relatives during patient care	437	4.40	0.78	272	3.14	1.41
9. Giving information suitable to the patients’ qualification, cultural background, cognitive state	438	4.58	0.70	288	3.25	1.37
10. Fully informing patients about their diseases	437	4.51	0.73	284	3.35	1.39
11. Establishing long term “partnerships” with patients (mostly with chronical diseases)	437	4.41	0.85	275	3.13	1.42
12. Handling patients as equals and with respect	437	4.88	0.41	302	3.96	1.25
13. An ongoing positive and motivated approach to work	436	4.65	0.58	347	3.72	1.12
14. The individual problem-solving skills (creativity) during everyday work	437	4.64	0.60	376	3.85	1.02
15. Handling appropriately patients’ expectations on therapy	434	4.02	0.87	277	3.14	1.35
16. Ability to work as a member of a team (in everyday situations)	438	4.50	0.69	327	3.56	1.20
17. Handling conflicts within the educational team and with the patients (and relatives)	436	4.61	0.62	280	3.03	1.33
18. Good time management	437	4.55	0.66	325	3.19	1.24
19. Improving emotional intelligence	436	4.37	0.79	285	3.04	1.29
20. Work-life balance	436	4.57	0.70	263	2.60	1.34
21. Information about carrier opportunities	438	4.00	0.90	281	2.87	1.33
22. Participation in further educational courses	438	4.35	0.76	292	3.24	1.36
23. Using assertive communication skills	436	3.88	0.92	274	3.03	1.31
24. Improving social intelligence	436	4.19	0.85	293	3.25	1.28

The table shows the importance and delivery of learning outcomes form the teachers’ perspective [mean importance: (n_max_ = 438, n_min_ = 436^a^), delivery (n_max_ = 420, n_min_ = 263)] and the standard deviations. The analysis shows that the importance of learning outcomes is rated all higher than their rate of delivery

^a^ The n_min_ shows the number of teachers who elvaluated all of the aspects, the n_max_ shows the number of teachers who evaluated at least one aspect

In case of the pedagogical skills, the nine selected items remained in the surveys as the expert committee qualified all of them essential according to the CVRs.

In the fifth phase, the committee reviewed the translated and the original versions as well to get semantic, idiomatic, experiential and conceptual equivalence [28–30], because with the help of the different language variations we were able to ask students of the English and German programs, coming from more than 60 different countries in the world. For the assurance of the linguistic validity of the questionnaire we used the back-translation technique, so the questionnaire was translated back from the target language into the original language by two independent translators.

In the sixth phase, we tested the face validity of the item pool. With this phase we aimed at testing the clarity of the wording of the questions, items in the questionnaire. Fifteen teachers and sixty students were asked to participate in this phase of our study at the University of Pécs, Medical School. We received comments on usage of words in some cases and we made some items more understandable with using examples. Finally, after the content and face validity process, in the final phase, we tested the Cronbach’s alpha as well. According to its values the items have acceptable internal consistency (i.e. Cronbach’s alpha, see Table 2).

**Table 2:** The estimates of internal consistency reliability (Cronbach’s alpha) of teacher’s the student’s questionnaire (sample)

Teachers (*N* = 15)		Students (*N* = 60)			
Items of	Cronbach’s alpha	Items of	Cronbach’s alpha		
			English (*N* = 20)	Hungarian (N = 20)	German (N = 20)
the importance of learning outcomes	0.907	the importance of learning outcomes	0.984	0.932	0.970
the delivery of learning outcomes	0.937	the question to what extent teachers have pedagogical skills	0.947	0.922	0.937
the need for further development of pedagogical skills	0.960	the need for further development of pedagogical skills	0.924	0.923	0.912

Table shows the estimates of internal consistency reliability (Cronbach’s alpha) for each of the item groups in the teacher’s and all the three languages version of the student’s questionnaire in the sample. According to its values the items have acceptable internal consistency

Table 3 presents the estimates of internal consistency reliability (Cronbach’s alpha) for each of the item groups (question numbers) in the teacher’s and all the three languages versions of the student’s questionnaire. The minimum of the Cronbach’s alpha values is 0.820 and all the others are higher than 0.9 suggesting a good-to-excellent level of reliability.

**Table 3:** The estimates of internal consistency reliability (Cronbach’s alpha) of teacher’s and student’s questionnaire

Teachers (*N* = 439)		Students			
Items of	Cronbach’s alpha	Items of	Cronbach’s alpha		
			English (*N* = 334)	Hungarian (*N* = 982)	German (*N* = 199)
the importance of learning outcomes	0.912	the importance of learning outcomes	0.973	0.901	0.907
the delivery of learning outcomes	0.963	the question to what extent teachers have pedagogical skills	0.935	0.889	0.896
the need for further development of pedagogical skills	0.918	the need for further development of pedagogical skills	0.926	0.869	0.889

Table shows the estimates of internal consistency reliability (Cronbach’s alpha) for each of the item groups in the teacher’s and all the three languages version of the student’s questionnaire in all surveyed data. According to its values the items have acceptable internal consistency

### Participants, sample

Based on the characteristics of the exploratory research, we strived to have filled out questionnaires in a boarder group of teachers and students that is the reason we sent out the questionnaires to the students having active legal status (13464) and teachers (1790) taking part in medical education in Hungary. We sent out the questionnaires two times in case of the students. In the beginning of November students get the questionnaire from the Dean’s Offices of the Hungarian Universities and in the middle of November from the Students’ Councils using social media as well. The response rates are 11.18% in case of the students (1505 students coming from more than 60 different countries [gender: male 551, female 952, not specified 2; language program: Hungarian 985, English 312, German 202; module: basic 727, preclinical 241, clinical 363, rotational year 157, not specified 27]) and 24.53% in case of the teachers (439 teachers [gender: male 248, female 190, not specified 1; teaching in the basic 235, clinical module 197, not specified 7; teaching experience in years: average 16.9, std. deviation 11.4, range 34.5, not specified 2]). Ethical approval for this study was granted by the Regional Ethical Committee of the University of Pécs. Missing data were not considered during the statistical analysis.

### Learning outcomes

Teachers rated the learning outcomes on a Likert-scale regarding the fact whether they are important to the doctor’s profession (rating scale: 1 = the least important, 5 = the most important) and to what extent they deliver them during the courses (rating scale: 1 = not at all, 5 = greatly).

### Pedagogical skills

Regarding pedagogical knowledge and skills, we used nine pedagogical skills for the teacher’s profession (listed in Table 4) based on Hegyi’s, Ballér’s grouping [31, 32] and the results and experiences of the preliminary interviews carried out with teachers as well as the pilot research about the comprehensibility of the questionnaire.

**Table 4:** Teachers’ and students’ view on and comparison (t-test) about the need for further development of pedagogical skills

Pedagogical skills	According to teachers		According to students		Significance of the difference between the means
	Mean	Standard Deviation	Mean	Standard Deviation	
Didactic knowledge	3.48	1.15	3.83	1.23	p < 0.01
Organizing and leading the learning process	3.28	1.23	3.92	1.16	p < 0.01
Psychological knowledge	3.21	1.19	3.56	1.20	p < 0.01
Communication knowledge	3.15	1.23	3.76	1.16	p < 0.01
Professional knowledge	3.12	1.16	2.30	1.21	p < 0.01
Improving adapting skills (e.g. flexibility in education considering the needs and expectations of the students and patients)	2.85	1.16	3.71	1.18	p < 0.01
Decision- making skills and rapid assessment of the situation	2.71	1.23	2.97	1.24	p < 0.01
Learning the ability of professional cooperation	2.47	1.23	3.24	1.30	p < 0.01
Empathy	2.37	1.24	3.63	1.23	p < 0.01

Table shows the opinions of students and teachers about the necessity of developing on the nine surveyed pedagogical fields. Students’ opinion compared to the opinions of teachers are all statistically higher according to the results of the t-tests (p < 0.01). (mean, nL = 436, nS = 1472)

Teachers were asked to rank their intention of improvement of their skills on the nine specified pedagogical fields. In addition, students were asked to rate the areas where teachers need further development. The ratings were performed on a 5-point scale as in the case of the learning outcomes (rating scale: 1 = not at all, 5 = greatly.)

### Statistical analysis

For data analysis gap matrices and independent sample t-tests (two-sided) were performed. Gap analysis matrix is a relationship matrix showing the gap between averages of two different factors, with one axis for the perceived importance and one for the delivery of a learning outcome. Application of the gap matrix in this study is used for demonstrating the difference between the importance of a learning outcome and the rate of delivery either in a negative or a positive way. The negative gap, when learning outcomes are under the diagonal, means that a learning outcome is rated more important than it is taught, so action is recommended to fulfil the needs of the respondents. On the contrary, the positive gap means that a learning outcome is delivered over the perceived needs (taught more by the teachers than its rate of importance). A zero gap or an optimal performance means that there is a balance between the two axes that is the importance and delivery of a specific learning outcome.

## Results

### Attitudes towards the importance of learning outcomes in relation to physicians’ profession and their rate of delivery based on the teachers’ answers

**Fig. 1:**
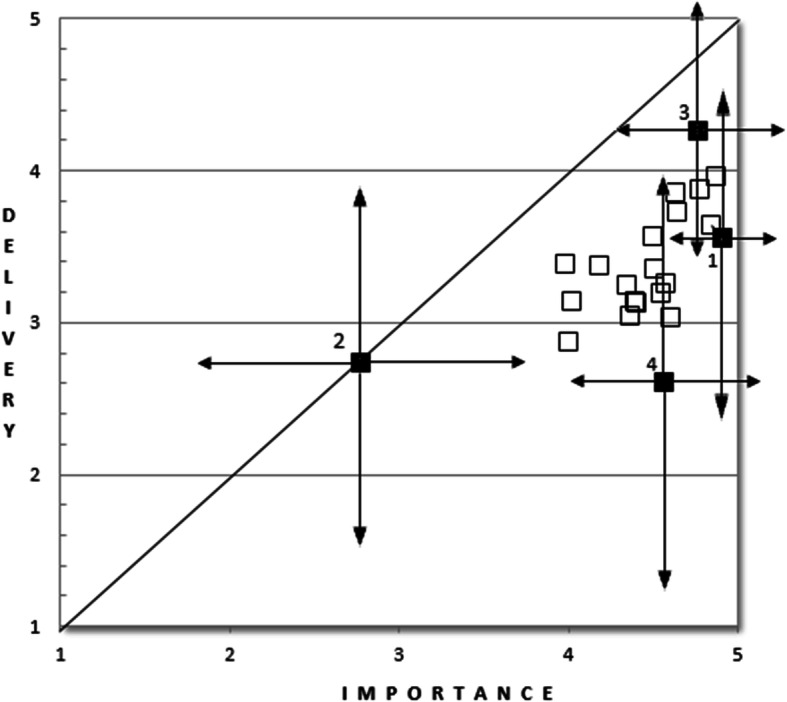
Gap of the perceived importance and delivery of the learning outcomes according to the teachers. The matrix shows that all the learning outcomes are rated more important than its rate of delivery by the teachers [mean; importance: (n_max_ = 438, n_min_ = 436) (The n_min_ shows the number of teachers who evaluated all of the aspects, the n_max_ shows the number of teachers who evaluated at least one aspect.); delivery: (n_max_ = 420, n_min_ = 263)]. The arrows show the standard deviation of the ratings of importance and of delivery for the given learning outcome. The mean ratings and the standard deviation of the following items are shown in parentheses. Maximum of importance: The professional experience needed for everyday work (4.91; 3.55, difference: -1,36). Minimum of importance and on the diagonal: The knowledge of historical overview of the medical disciplines (2.78; 2.73, difference: -0,05). Maximum of delivery: The timely theoretical and practical knowledge to the everyday work (4.77; 4.26, difference: -0,51). Minimum of delivery: Work-life balance (4.57; 2.60, difference: -1,97)

As shown in Fig. 1 and Table 1, the teachers in this study rated twenty of the important learning outcomes with an average higher than 4 and only four with an average equal with or lower than 4.

This analysis points out that teachers are mostly aware of the fact that learning outcomes would play an important role in performing the everyday professional work of a physician (as they rated them with high important rates), but they feel they are not conveying them at the appropriate level of importance. The teachers ranked the professional practice needed for everyday work (4.91) as the most important learning outcome, which has an average of 3.55 regarding its delivery. So, it is under the diagonal, showing a negative gap. Even in the case of the least important learning outcome (the knowledge of historical overview of the medical disciplines – 2.78), we can observe a negative gap; a higher rate of importance than delivery (2.73). With respect to the low difference between the two rates (0.05), this learning outcome seems to appear on the diagonal of the gap matrix. This finding can be observed in the cases of the most and least delivered learning outcomes as well. To sum it up, no positive gap can be observed in Fig. 1, suggesting that the learning outcomes are ranked by the teachers more important than they deliver them during the courses and teachers do not meet their own expectations during teaching.

### Attitudes towards the need for further development of the pedagogical skills of teachers based on the teachers’ and students’ answers

Both teachers’ and students’ ratings were analyzed regarding the need for further development in pedagogical skills. As shown in Table 4, teachers rated the didactic knowledge (3.48), organizing and leading the learning process (3.28) as well as psychological knowledge (3.21) as the skills requiring further development. Empathy (2.37), learning the ability of professional cooperation (2.47), decision-making skills (2.71) and improving adapting skills (2.85) received the lowest ratings from teachers.

Regarding the ratings of students, we observed that the highest averages appeared in the following fields: organizing and leading the learning process (3.92), didactic knowledge (3.83) and communication knowledge (3.76). Altogether, there were six factors that had averages higher than 3.5. The lowest ratings can be seen in the cases of the following pedagogical skills: professional knowledge (2.30), decision-making skills (2.97) and improving adapting skills (3.71) and learning the ability of professional cooperation (3.24).

The opinions of students about the necessity of developing on the nine surveyed pedagogical fields compared to the opinions of teachers are all statistically higher according to the results of the t-tests (*p* < 0.01). The opinions of students about the necessity of developing on the nine surveyed pedagogical fields in the different language groups differ significantly from each other. The students of the English program ranked the need for professional knowledge and decision-making skills at the highest level. There were three factors (empathy and learning the ability of professional cooperation, improving adapting skills) which were ranked by students of the German program lower than the other students. In the other four factors the Hungarian students ‘average score was higher than in case of the other language groups.

## Discussion

Our study shows the importance of learning outcomes in relation to the rate of delivery: teachers rated the importance of learning outcomes and rated how far they deliver them during their courses. Using gap matrix analysis, our hypotheses were confirmed because teachers do not deliver the analyzed learning outcomes as much as their importance and they are not as aware of the importance of learning pedagogical skills as students estimated they should.

### Learning outcomes

Teachers rated all 24 learning outcomes as more important (4.39 in average) in the performance of the profession of doctors than their rates of delivery (3.29 in average). The fact that teachers rate the efficient delivery of the learning outcomes of physicians between the average of 3.00 and 4.00 suggests that teachers critically evaluate their teaching performance. Our study points out that only one learning outcome (the knowledge of historical overview of the medical disciplines) is nearly on the diagonal, meaning a “balance” between the estimated importance and actual delivery, which is insufficient to give a high quality of medical education. The results also suggest that teachers have high expectations, because they ranked the learning outcomes with high importance and think that it would be useful in the everyday job of a doctor, but they were not satisfied with their results in terms of delivery. Dissatisfaction of the teachers may have roots either in.

the fact that teachers do not want to deliver learning outcomes at a high quality level or they do not have enough pedagogical knowledge or time to prepare for teaching because of their other duties (e.g. participation in health care and scientific research). The lack of pedagogical knowledge and teaching methods [10–12, 33] and the fact that a different teaching method [2, 34] can give different results in terms of the delivery of learning outcomes as it is stated in Krupat’s and White’s articles [2, 35] can also be reasons for the lower rates. Although Steinert’s [34] finding supports the initiatives designed to enhance teaching performance, in our research it seems that teachers do not think that improving their pedagogical skills can play an important role in being more efficient in teaching and narrow the gap between their and the students’ expectations. Our results reflect the Hungarian practice, which lacks pedagogical courses for teachers and does not involve the quality of teaching in career as valuable as research activity is valued.

### Pedagogical skills

There are striking differences in the ratings of teachers and students regarding pedagogical skills that require further investigations to find the specific reasons.

The ratings given by teachers for pedagogical skills indicate a relatively low interest in further development. Out of the nine pedagogical skills, only one is an exception, as the average for the need for further development was higher among teachers than students: the professional knowledge. This result does not support the paradigm shift from teacher to student-oriented learning, teaching method [18]. Furthermore, we would like to point out that students rated the need for professional knowledge with the lowest average. It suggests that they feel it the least crucial field for the further development of teachers. As a reason for having high teachers’ rates can be that developing professional skills is a part of their everyday work as a doctor and highly valued in the carrier ladder. The other skills providing the basis for delivering knowledge, among others professional knowledge, were all rated higher by students (independently from the language of the program) than by teachers, so students recommend further development more than teachers think they should have. Critical evaluation of the teachers can be derived from the fact that medical teachers have not had pedagogical courses preparing them to teach [8] and their teaching skills are based on their experience as learners [9]. In Hungary, the prerequisite of being a teacher in Medical Schools does not include the completion pedagogical courses. Besides, education has deep roots in traditional pedagogical methods and using non-traditional methods can require huge investments (such as infrastructural) and require a paradigm shift not only in methods, but in thinking, in health care protocols, in rules, in communication etc. One of the symposia, held during the 2019 conference of the Association for Medical Education in Europe (AMEE) entitled “How to train your dragon”, dealt with the difficulties of paradigm shift needs to be done in teaching attitude to cope with the common enemy; a concern over the preparedness of students working in increasingly complex health care settings and the ability of medical schools to adapt to these demands. According to the presenters, faculty development opportunities (including pedagogical courses for medical teachers) are fundamental for the cultural transformations needed to tackle these challenges. These questions are challenging and have to be answered in the near future [36].

Furthermore, using pedagogical skills and methods during the courses are considered as a method mostly used in primary schools and not in higher education based on semi-structured interviews performed in the research. To put it in another way, in line with the results of previous studies [9, 12, 14] it is not enough to be educated in the medical doctor’s profession, teachers should also know how to teach the profession effectively.

### Limitations

While analyzing the results, we have to take into account that the pattern of the rankings of teachers’ pedagogical skills suggests that their ratings were influenced by their everyday teaching routine, the way how they became teachers (without any pedagogical courses [8, 9]) and they may not be aware of their needs for further development [37]. Teachers who were taught by doctors during their own studies and became teachers based on their experience can have only a poor knowledge of pedagogical concepts, so another confounder can be that no glossary terms were attached to the questionnaires. Therefore, the comprehension of teachers and students as to the learning outcomes and pedagogical skills may contribute to the lack of concordance between the ratings.

### Generalizability

Our findings have potentially important implications for changing the attitudes in teaching future physicians in Hungary and in those countries where non-traditional teaching methods are rarely used. First, to the best of our knowledge there has not been any questionnaire in the world analyzing the attitudes of teachers towards the learning outcomes and of teachers and students towards the pedagogical skills in Hungarian medical education. Second, our research and other studies such as the one of Hesketh et al. [14] reveal that students would be delighted if they could get a higher quality of education, especially regarding learning outcomes. Third, our research suggests that teachers may not recognize that learning pedagogical skills can have a huge contribution to the success of teaching and performance of students, besides professional medical knowledge as it is supported by Steinert as well [34]. The discrepancy between the rankings of teachers and students may have an impact on launching new pedagogy-based courses for teaching personnel. Fourth, the study reveals the need for a discourse between teachers and students and their expectations regarding teaching, professional techniques, method and curriculum development. Fifth and finally, the need for a teaching personnel capable of self-development in pedagogy is implied in the study, as well as building a system of special pedagogical education in medical education in Hungary. Moreover, the motivation for changing the teaching methods is being accelerated and forced by the appearance of COVID-19, since teachers have to adapt the new circumstances regarding distance (remote) learning which evokes new, creative ideas and solutions for teaching in a different way. These methods will not disappear after COVID-19 and a new, exciting period will start in medical higher education.

Although the study points out and international research outcomes also support the necessity for the further pedagogical development of teachers to deliver learning outcomes on higher level, there is a no published evidence suggesting the same expectation of the patients who are one of the major participants in healthcare. Due to the lack of this evidence and because the analysis is based only on the teachers’ and students’ opinions more evidence on the subject is needed.

## Conclusions

The study underlines the results of previous studies so points to the necessity of a transition and paradigm shift in medical education from sharing exclusively professional knowledge to pedagogically prepared practice, patient and student oriented teaching methods (non-traditional teaching methods) in Hungarian medical education as well as emphasizing the acquisition of pedagogical skills as part of the training of medical teachers.

## Supplementary Information

**Additional file 1.** Attitudes of teachers towards the importance, the rate of acquisition, the delivery of learning outcomes and the need for further development of pedagogical skills: a national survey. The questionnaire was designed to examine the attitudes of teachers towards learning outcomes of medical students in the four Hungarian higher education institution offering medical education to get a view about the opinions about their importance and rate of delivery as well as to analyze the pedagogical skills of teachers from the teachers’ perspective.

**Additional file 2.** Attitudes of students towards the importance, the rate of acquisition of learning outcomes and the need for further development of pedagogical skills: a national survey. The questionnaire was designed to examine the attitudes of students towards learning outcomes of medical students in the four Hungarian higher education institution offering medical education to get a view about the opinions about their importance and rate of acquisition as well as to analyze the pedagogical skills of teachers from the students’ perspective.

## Data Availability

The datasets used and/or analysed during the current study are available from the corresponding author on reasonable request.
