# Supplementary material for: Do we need special pedagogy in medical schools? – Attitudes of teachers and students in Hungary: a cross-sectional study
Source: BMC Med Educ. 2020 Nov 26;20:472. doi: 10.1186/s12909-020-02385-x (PMC7689636; doi:10.1186/s12909-020-02385-x)
Supplement: Supplementary file 1 — Additional file 1. Attitudes of teachers towards the importance, the rate of acquisition, the delivery of learning outcomes and the need for further development of pedagogical skills: a national survey. The questionnaire was designed to examine the attitudes of teachers towards learning outcomes of medical students in the four Hungarian higher education institution offering medical education to get a view about the opinions about their importance and rate of delivery as well as to analyze the pedagogical skills of teachers from the teachers’ perspective. [file 12909_2020_2385_MOESM1_ESM.docx]

**Additional file 1.**

Attitudes of teachers towards the importance, the rate of acquisition, the delivery of learning outcomes and the need for further development of pedagogical skills: a national survey

*ANSWERING IS VOLUNTARY!*

QUESTIONNAIRE

Dear Colleague!

The quality of education is essential in the life of our Faculty. We would like to get to know your opinion to be able to modernize our education and to meet the latest professional challenges. Giving answers for all our questions you can give a huge help for us to realize our goals. The questionnaire is anonymous. In spite of the fact that personal data is not included in the questionnaire, we treat all the answers confidential and publicize only in a summarized way. Completing the questionnaire takes approximately 10 minutes. The results of the survey will be available on the website of the Faculty.

Please press on the "küldés" button at the end of the survey to complete the questionnaire.

*Thank you for your cooperation.*

dean

1. Which Faculty do you have legal status?

| University of Debrecen Faculty of Medicine | 1 |
| --- | --- |
| University of Pécs Medical School | 2 |
| Semmelweis University Faculty of Medicine | 3 |
| University of Szeged Faculty of Medicine | 4 |

1. What is your main task?

| teaching |  |
| --- | --- |
| doing research |  |
| patient care |  |

1. What is your position?

| colleague | 1 |
| --- | --- |
| leader | 2 |
| top leader | 3 |

1. Where do you work?

| Department | 1 |
| --- | --- |
| Clinic | 2 |

1. Since when have you been working at the faculty?

| less than a year | 1 |
| --- | --- |
| 1-5 years | 2 |
| 6-10 years | 3 |
| 11-20 years | 4 |
| 21-30 years | 5 |
| more than 30 years | 6 |

1. Sex:

| male |  |
| --- | --- |
| female |  |

| ***How important do you consider the following to perform your every day job as a doctor?*** | **the least important** | **2** | **3** | **4** | **the most important** |
| --- | --- | --- | --- | --- | --- |
| 1. The timely theoretical and practical knowledge to the everyday work | O | O | O | O | O |
| 1. The professional practice needed for the everyday work | O | O | O | O | O |
| 1. The knowledge of historical overview of the medical disciplines | O | O | O | O | O |
| 1. The flexible professional and everyday thinking | O | O | O | O | O |
| 1. Respecting human dignity of the patients and the relatives during patient care | O | O | O | O | O |
| 1. Respecting the different demographic (sex, age), social and economic characteristics during patient care | O | O | O | O | O |
| 1. Respecting individual specialty during patient care (e.g. familiar background, emotional state, sexual orientation) | O | O | O | O | O |
| 1. Treating the emotional reactions of the patients and the relatives during patient care | O | O | O | O | O |
| 1. Giving information suitable to the patients’ qualification, cultural background, cognitive state | O | O | O | O | O |
| 1. Fully informing patients about their diseases | O | O | O | O | O |
| 1. Establishing long term “partnerships” with patients (mostly with chronical diseases) | O | O | O | O | O |
| 1. Handling patients as equals and with respect | O | O | O | O | O |
| 1. An ongoing positive and motivated approach to work | O | O | O | O | O |
| 1. The individual problem-solving skills (creativity) during everyday work | O | O | O | O | O |
| 1. Handling stress and avoiding burnout | O | O | O | O | O |
| 1. Handling appropriately patients’ expectations on therapy | O | O | O | O | O |
| 1. Ability to work as a member of a team (in everyday situations) | O | O | O | O | O |
| 1. Handling conflicts within the educational team and with the patients (and relatives) | O | O | O | O | O |
| 1. Good time management | O | O | O | O | O |
| 1. Improving emotional intelligence | O | O | O | O | O |
| 1. Work-life balance | O | O | O | O | O |
| 1. Information about carrier opportunities | O | O | O | O | O |
| 1. Participation in further educational courses | O | O | O | O | O |
| 1. Using assertive communication skills | O | O | O | O | O |
| 1. Improving social intelligence | O | O | O | O | O |

| ***8. To what extent do students learn the following during their studies?*** | **Not at all** | **2** | **3** | **4** | **Greatly** |
| --- | --- | --- | --- | --- | --- |
| 1. The timely theoretical and practical knowledge to the everyday work | O | O | O | O | O |
| 1. The professional practice needed for the everyday work | O | O | O | O | O |
| 1. The knowledge of historical overview of the medical disciplines | O | O | O | O | O |
| 1. The flexible professional and everyday thinking | O | O | O | O | O |
| 1. Respecting human dignity of the patients and the relatives during patient care | O | O | O | O | O |
| 1. Respecting the different demographic (sex, age), social and economic characteristics during patient care | O | O | O | O | O |
| 1. Respecting individual specialty during patient care (e.g. familiar background, emotional state, sexual orientation) | O | O | O | O | O |
| 1. Treating the emotional reactions of the patients and the relatives during patient care | O | O | O | O | O |
| 1. Giving information suitable to the patients’ qualification, cultural background, cognitive state | O | O | O | O | O |
| 1. Fully informing patients about their diseases | O | O | O | O | O |
| 1. Establishing long term “partnerships” with patients (mostly with chronical diseases) | O | O | O | O | O |
| 1. Handling patients as equals and with respect | O | O | O | O | O |
| 1. An ongoing positive and motivated approach to work | O | O | O | O | O |
| 1. The individual problem-solving skills (creativity) during everyday work | O | O | O | O | O |
| 1. Handling stress and avoiding burnout | O | O | O | O | O |
| 1. Handling appropriately patients’ expectations on therapy | O | O | O | O | O |
| 1. Ability to work as a member of a team (in everyday situations) | O | O | O | O | O |
| 1. Handling conflicts within the educational team and with the patients (and relatives) | O | O | O | O | O |
| 1. Good time management | O | O | O | O | O |
| 1. Improving emotional intelligence | O | O | O | O | O |
| 1. Work-life balance | O | O | O | O | O |
| 1. Information about carrier opportunities | O | O | O | O | O |
| 1. Participation in further educational courses | O | O | O | O | O |
| 1. Using assertive communication skills | O | O | O | O | O |
| 1. Improving social intelligence | O | O | O | O | O |

| ***9. To what extent do you deliver the following during your courses?*** | **Not at all** | **2** | **3** | **4** | **Greatly** |
| --- | --- | --- | --- | --- | --- |
| 1. The timely theoretical and practical knowledge to the everyday work | O | O | O | O | O |
| 1. The professional practice needed for the everyday work | O | O | O | O | O |
| 1. The knowledge of historical overview of the medical disciplines | O | O | O | O | O |
| 1. The flexible professional and everyday thinking | O | O | O | O | O |
| 1. Respecting human dignity of the patients and the relatives during patient care | O | O | O | O | O |
| 1. Respecting the different demographic (sex, age), social and economic characteristics during patient care | O | O | O | O | O |
| 1. Respecting individual specialty during patient care (e.g. familiar background, emotional state, sexual orientation) | O | O | O | O | O |
| 1. Treating the emotional reactions of the patients and the relatives during patient care | O | O | O | O | O |
| 1. Giving information suitable to the patients’ qualification, cultural background, cognitive state | O | O | O | O | O |
| 1. Fully informing patients about their diseases | O | O | O | O | O |
| 1. Establishing long term “partnerships” with patients (mostly with chronical diseases) | O | O | O | O | O |
| 1. Handling patients as equals and with respect | O | O | O | O | O |
| 1. An ongoing positive and motivated approach to work | O | O | O | O | O |
| 1. The individual problem-solving skills (creativity) during everyday work | O | O | O | O | O |
| 1. Handling stress and avoiding burnout | O | O | O | O | O |
| 1. Handling appropriately patients’ expectations on therapy | O | O | O | O | O |
| 1. Ability to work as a member of a team (in everyday situations) | O | O | O | O | O |
| 1. Handling conflicts within the educational team and with the patients (and relatives) | O | O | O | O | O |
| 1. Good time management | O | O | O | O | O |
| 1. Improving emotional intelligence | O | O | O | O | O |
| 1. Work-life balance | O | O | O | O | O |
| 1. Information about carrier opportunities | O | O | O | O | O |
| 1. Participation in further educational courses | O | O | O | O | O |
| 1. Using assertive communication skills | O | O | O | O | O |
| 1. Improving social intelligence | O | O | O | O | O |

| *10. To what extent do you think you need further development in the following skills?* | **Not at all** | **2** | **3** | **4** | **Greatly** |
| --- | --- | --- | --- | --- | --- |
| 1. Professional knowledge | O | O | O | O | O |
| 1. Didactic knowledge | O | O | O | O | O |
| 1. Communicational knowledge | O | O | O | O | O |
| 1. Psychological knowledge | O | O | O | O | O |
| 1. Organizing and leading the learning process | O | O | O | O | O |
| 1. Improving adapting skills (e.g. flexibility in education considering the needs and expectations of the students and patients) | O | O | O | O | O |
| 1. Decision making skills and rapid assessment of the situation | O | O | O | O | O |
| 1. Empathy | O | O | O | O | O |
| 1. Learning the ability of professional cooperation | O | O | O | O | O |

| 1. ***What do you think in which areas would be needed further education for the lecturers in your Faculty?*** | **Not at all** | **2** | **3** | **4** | **Greatly** |
| --- | --- | --- | --- | --- | --- |
| 1. Professional knowledge | O | O | O | O | O |
| 1. Didactic knowledge | O | O | O | O | O |
| 1. Communicational knowledge | O | O | O | O | O |
| 1. Psychological knowledge | O | O | O | O | O |
| 1. Organizing and leading the learning process | O | O | O | O | O |
| 1. Improving adapting skills (e.g. flexibility in education considering the needs and expectations of the students and patients) | O | O | O | O | O |
| 1. Decision making skills and rapid assessment of the situation | O | O | O | O | O |
| 1. Empathy | O | O | O | O | O |
| 1. Learning the ability of professional cooperation | O | O | O | O | O |

1. If you have any comments regarding the questionnaire, please don't hesitate to share with us.

……………………………………………………………………………………………………………………………………………………………………………………………………………………………………………………………………………………………………………………………………………………………………………………………………………………………..

Thank you for your cooperation!
